# Hitting pause on chemotherapy-induced alopecia: transient p53 activation as a guardian of the hair follicle

**DOI:** 10.1172/JCI205966

**Published:** 2026-05-01

**Authors:** Edward B. Li, Meredith Klay, Rui Yi

**Affiliations:** 1 Department of Dermatology, Northwestern University Feinberg School of Medicine, Chicago, Illinois, USA.; 2 Hair-Alopecia Innovation and Research Program, Northwestern Medicine, Chicago, Illinois, USA.; 3 Department of Pathology,; 4 Driskill Graduate Program in Life Sciences, and; 5 Robert H. Lurie Comprehensive Cancer Center, Northwestern University Feinberg School of Medicine, Chicago, Illinois, USA.

## Abstract

Chemotherapy-induced alopecia (CIA) is a common and highly visible adverse effect of chemotherapy with substantial psychosocial and quality-of-life burdens. In this issue, Gherardini and colleagues described a targeted strategy to prevent CIA using ALRN-6924, a stapled peptide that transiently activates p53 and induces cell cycle arrest in proliferating TP53 wild-type tissues, such as the hair follicle. In ex vivo human scalp hair follicle culture, ALRN-6924 protected matrix keratinocytes and bulge stem cells from paclitaxel- and cyclophosphamide-induced injury, reducing apoptosis, DNA damage, and other pathologic features. These findings nominate precision chemoprotection as a promising supportive care approach for mitigating CIA.

Chemotherapy-induced alopecia (CIA) is one of the most outwardly visible and psychologically devastating side effects of systemic cytotoxic chemotherapies (1, 2). For many patients, hair loss can often shape their oncology experience, treatment satisfaction, and quality of life in clinically meaningful ways (3, 4). As cancer survivorship continues to improve, the dermatology and oncology communities are increasingly recognizing persistent or permanent alopecia following certain chemotherapy regimens, further adding to the disfigurement and psychologic distress of patients during an already challenging time (5).

The hair follicle’s vulnerability to cytotoxic chemotherapy is rooted in its inherent biology as one of the most highly proliferative tissues in human body (Figure 1A). During anagen hair growth, rapidly dividing matrix keratinocytes in the hair bulb fuel hair shaft production, while epithelial hair follicle stem cells (eHFSCs) in the bulge preserve the regenerative capacity required for subsequent cycles of regression and regrowth (6). Because cytotoxic chemotherapies target highly proliferative cells, the growing hair follicle ranks among the most chemotherapy-sensitive tissues in the body (7), yet clinical options to mitigate this toxicity remain strikingly limited (8). Scalp cooling is currently the only FDA-approved intervention for CIA prevention. Although it can reduce the frequency and severity of hair loss in selected patients, scalp cooling is not universally effective, can be challenging to implement, and remains logistically and financially inaccessible in many practice settings.

The current study by Gherardini and colleagues (9) is thus notable because it advances a different conceptual framework for CIA prevention. Rather than attempting to reduce chemotherapy exposure to the scalp, the authors seek to make the follicle itself resistant to chemotherapy damage through transient p53-mediated cell cycle arrest. Building on earlier work demonstrating that temporary G1 arrest with CDK4/6 inhibition can protect human hair follicles ex vivo (10), the authors tested ALRN-6924, a stapled peptide that activates p53 signaling by binding to its endogenous suppressors, MDM2 and MDMX, and preventing them from degrading p53 (11). At lower doses, this agent was previously shown to activate p53 and induce p21-dependent cell cycle arrest in normal tissue harboring wild-type TP53, while avoiding protection of cancer cells that harbor TP53 mutations (11). Moreover, in a screen of wild-type and mutant TP53 cancer cell lines, ALRN-6924 was highly active in 93% of wild-type TP53 cell lines but only in 2% of mutant TP53 cell lines, highlighting its specificity for wild-type TP53 activation (12). Leveraging this targeted selection, Gherardini et al. noted that roughly half of all cancers harbor TP53 mutations, including many gynecologic and breast cancers frequently treated with cytotoxic chemotherapies associated with transient and persistent CIA. This raises the possibility of using ALRN-6924 as a selective cytoprotective strategy for hair follicles around the time of chemotherapy administration to prevent CIA (Figure 1B).

## Hair follicle protection without follicular toxicity

Because the natural cycling of hair follicles from the growth phase to regression phase involves matrical keratinocyte cell cycle arrest and cell death, a key initial question in the feasibility of this study involves identifying whether the transient p53-mediated cell cycle arrest caused by ALRN-6924 would cause significant hair follicle cytotoxicity and catagen induction. In organ-cultured, full-length human scalp hair follicles, the authors established that the hair follicle matrix and eHFSC-containing bulge region exhibited robust p21 expression following ALRN-6924 treatment, as well as an accompanying decrease in cell proliferation as marked by Ki67, yet lacked a significant increase in apoptosis. Furthermore, the treated hair follicles remained in anagen phase with normal hair shafts and no pigmentary abnormalities, demonstrating that ALRN-6924 does not promote catagen entry (and thereby clinical hair shedding) or abnormal hair shaft production. By demonstrating that hair follicle transient activation of the p53/p21 axis can be achieved with ALRN-6924 application without overt toxicity in an ex vivo culture system, the authors provided a critical foundation to their subsequent chemotherapy protection studies, since follicular toxicity as a result of ALRN-6924 application alone would be counterproductive as a CIA prevention strategy.

## Hair matrix and eHFSC protection from chemotherapy-induced injury

The authors then tested whether transient p53-mediated cell cycle arrest reduced hair follicle cytotoxicity from paclitaxel (PTX) and 4-hydroxycyclophosphamide (4-HC), two commonly used cytotoxicity chemotherapies in the treatment of gynecologic and breast malignancies. Treatment of ex vivo hair follicles with PTX induced matrix injury, including apoptosis, mitotic catastrophe, micronucleation, and pigmentary abnormalities, all of which were significantly reduced by pretreatment with ALRN-6924, suggesting that transient cell cycle arrest in the hair matrix protects from PTX-associated cytotoxic effects. Treatment with 4-HC yielded similar results, with the additional finding that the treatment promoted premature catagen entry. Pretreatment with ALRN-6924 reduced this premature regression while reducing apoptosis and follicular damage as well, further suggesting that this targeted mechanism could serve to reduce the severity of CIA.

Perhaps the most clinically important component of this study is the focus on the bulge eHFSC compartment. Persistent CIA has been hypothesized to reflect hair follicle injury beyond the proliferative matrix to include damage to the permanent portion of the hair follicle, such as the eHFSCs and their defined tissue microenvironment niche (7). Damage to eHFSCs could deplete this important stem cell population and impair the hair follicle’s ability to complete subsequent hair cycles (13). In Gherardini et al.’s ex vivo hair follicle culture model, PTX and 4-HC treatment also increased apoptosis of and DNA damage to Keratin 15–positive (K15^+^) bulge eHFSCs and induced vimentin expression, together suggesting that these stem cells are also directly damaged by cytotoxic chemotherapies despite their relatively quiescent nature and can adopt a pathologic epithelial-mesenchymal transition phenotype that has previously been observed in inflammatory scarring alopecias, such as lichen planopilaris (14). Pretreatment of hair follicles with ALRN-6924 reduced DNA damage markers, apoptosis, and vimentin expression in K15^+^ bulge eHFSCs across both chemotherapeutic agents, supporting the interpretation that transient p53 activation protects not only the rapidly dividing matrical keratinocytes but also the long-lived quiescent hair follicle stem cell population. This expands the scope and applicability of this CIA prevention strategy to include not only transient but also persistent CIA as well, an unfortunate side effect for which many patients have expressed regret over undertaking a particular chemotherapy regimen in our clinical practice.

Gherardini and colleagues further solidified the mechanistic link between ALRN-6924, the p53/p21 axis, and the hair follicle protection observed by silencing p21 in ex vivo hair follicles. Successful knockdown of p21 abrogated the ALRN-6924 pretreatment protective effects against chemotherapy-induced keratinocyte DNA damage and cell death. This result substantially strengthens the mechanistic link between ALRN-6924 and the p53/p21 axis as the functional driver of the observed hair follicle protection phenotype.

## Topical delivery increases likelihood of clinical adoption

The authors extended their work on systemic ALRN-6924 pretreatment in microdissected hair follicles with a model of topical application using full-thickness human scalp skin organ culture (10). They formulated ALRN-6924 in a clinically applicable vehicle containing ingredients approved for topical minoxidil delivery and applied small volumes of this formulation to the surface of cultured scalp skin surfaces. In this model, topical application of ALRN-6924 robustly induced p21 upregulation in the hair follicle matrix and bulge eHFSCs, suggesting that ALRN-6924 reached these dermal and hypodermal sites with only topical drug application. Furthermore, topical pretreatment with ALRN-6924 was able to reduce the hair follicle keratinocyte toxicity associated with PTX treatment, suggesting that topical drug application could be sufficient in a clinical setting to reduce the risk and severity of CIA. However, further studies would be required to directly interrogate the penetration and delivery of stapled-peptide molecules to the hair follicle through topical application. The feasibility of a topically applied medication would allow a more targeted application of the medication to the scalp and other hair-bearing areas where hair preservation is desired during chemotherapy, boosting the real-world applicability of this therapy.

## Conclusion

This work advances CIA research by proposing a mechanistically targeted, biomarker-informed framework for hair follicle protection. By showing that transient activation of the p53/p21 axis can protect both hair matrix keratinocytes and bulge eHFSCs from chemotherapy injury ex vivo, Gherardini and colleagues expand the goal of CIA prevention beyond short-term hair retention to preservation of long-term follicular regenerative capacity. More broadly, the work illustrates a precision supportive care framework, in which tumor genotype may help guide selective protection of normal tissues, such as the gastrointestinal tract and bone marrow, during chemotherapies.

At the same time, the translational path remains uncertain. The ex vivo hair follicle organ culture time frame is sufficient for mechanistic discovery, but the true tolerance of the hair follicle matrix for cell cycle arrest for a longer time without progression into catagen remains to be determined. Further, human scalp hair follicles are long-lived mini-organs with anagen growth phases lasting years (15). Repeated transient p53 activation across multiple cycles of chemotherapy could influence growth and cycling characteristics and require longer term observations to verify. These concerns are reinforced by a prior phase Ib trial of ALRN-6924 in TP53-mutated, HER2-negative breast cancer, which was terminated after early data showed grade 4 neutropenia and alopecia after cycle 1 of treatment (ClinicalTrials.gov; NCT05622058). However, even if ALRN-6924 itself may not be the final solution, this study makes a compelling case that selective, time-limited chemoprotection could become an important direction for future CIA prevention without compromising the intended therapeutic goal of chemotherapy.

## Conflict of interest

The authors have declared that no conflict of interest exists.

## Funding support

This work is the result of NIH funding, in whole or in part, and is subject to the NIH Public Access Policy. Through acceptance of this federal funding, the NIH has been given a right to make the work publicly available in PubMed Central.

- NIH R01HD107841, R01AR081103, and R01AR071435 (to RY).
- Che Family Foundation (to EBL).
- Dr. Petra and Owen Rissman Foundation (to EBL).
- Ghandi Family Foundation (to EBL).
- NIH T32 Program in Cutaneous Biology 5T32AR060710 (to EBL).
- Dermatology Foundation Dermatologist-Investigator Research Fellowship (to EBL).
- Dermatology Foundation Physician-Scientist Career Developmental Award (to EBL).
- Che Family Foundation (to RY).
- Dr. Petra and Owen Rissman Foundation (to RY).

## Figures and Tables

**Figure 1:**
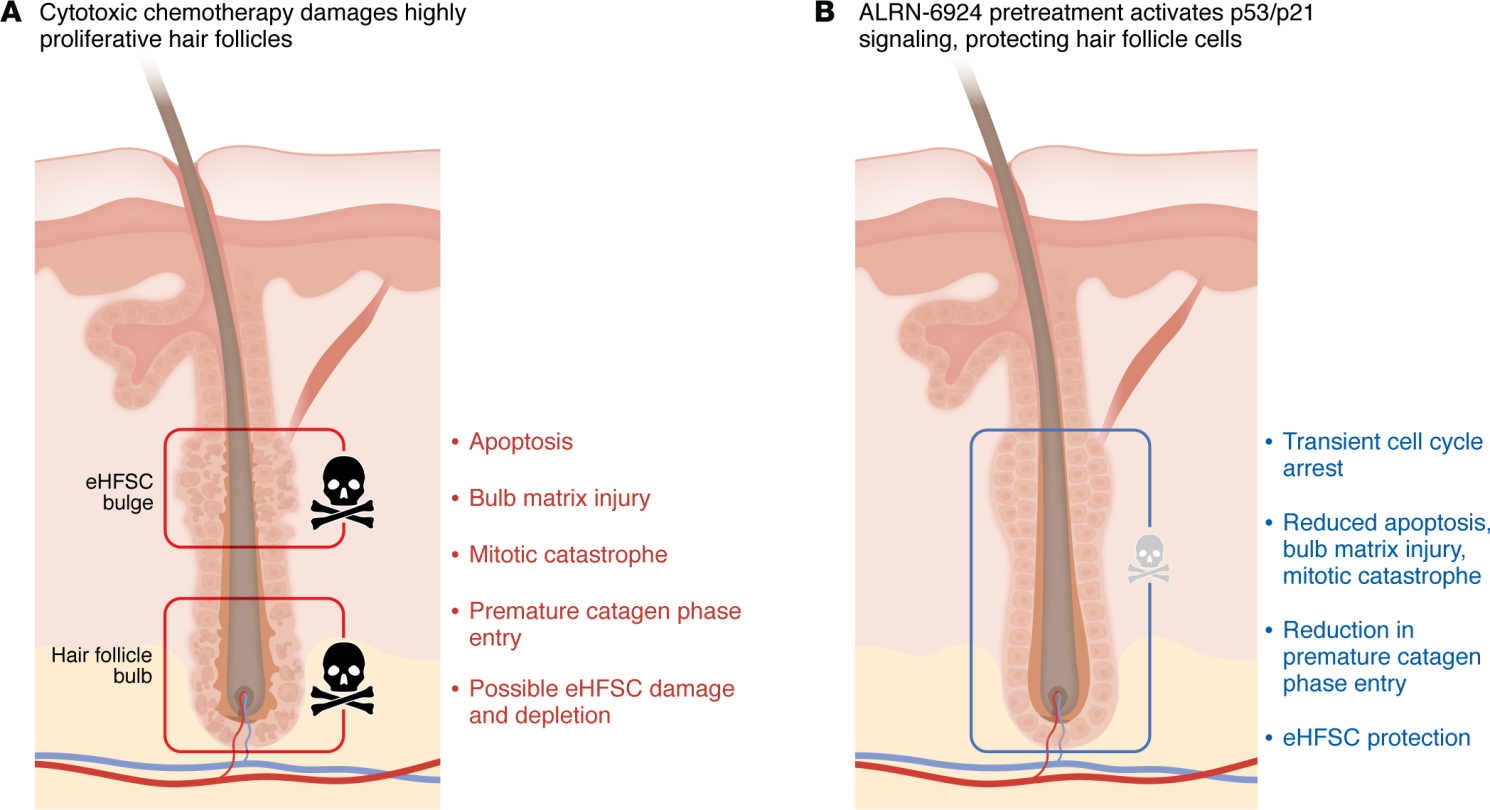
Protecting the hair follicle from chemotherapy-induced injury. (**A**) Chemotherapy-induced alopecia (CIA) is a highly visible adverse effect of systemic chemotherapies that is often distressing to patients. The hair follicle’s vulnerability to chemotherapy is attributed to the rapid proliferation of keratinocytes in the hair follicle bulb that occurs during the anagen phase of hair growth. Chemotherapy can also damage quiescent stem cells in the epithelial hair follicle stem cell (eHFSC) bulge, which may underlie persistent CIA. (**B**) Gherardini et al. (9) showed that pretreating ex vivo human hair follicles with ALRN-6924 protected hair follicles from CIA. Mechanistically, ALRN-6924 reactivated p53/p21 signaling, forcing a transient cell cycle arrest that reduced apoptosis and DNA damage in bulb keratinocytes and the eHFSC compartment. This strategy highlights the potential to develop precision chemoprotective treatments as supportive care approaches to prevent CIA.

## Version 1 05/01/2026

Electronic publication
